# Rare complications of multikinase inhibitor treatment

**DOI:** 10.20945/2359-3997000000090

**Published:** 2018-10-01

**Authors:** Fabián Pitoia, Angélica Schmidt, Fernanda Bueno, Erika Abelleira, Fernando Jerkovich

**Affiliations:** 1 Universidad de Buenos Aires University of Buenos Aires Division of Endocrinology Buenos Aires Argentina Division of Endocrinology, University of Buenos Aires, Buenos Aires, Argentina

**Keywords:** Multikinase inhibitors, rare adverse events, sorafenib, vandetanib, thyroid carcinoma

## Abstract

**Objective:**

The advent of multikinase inhibitor (MKI) therapy has led to a radical change in the treatment of patients with advanced thyroid carcinoma. The aim of this manuscript is to communicate rare adverse events that occurred in less than 5% of patients in clinical trials in a subset of patients treated in our hospital.

**Subjects and methods:**

Out of 760 patients with thyroid cancer followed up with in our Division of Endocrinology, 29 (3.8%) received treatment with MKIs. The median age at diagnosis of these patients was 53 years (range 20-70), and 75.9% of them were women. Sorafenib was prescribed as first-line treatment to 23 patients with differentiated thyroid cancer and as second-line treatment to one patient with advanced medullary thyroid cancer (MTC). Vandetanib was indicated as first-line treatment in 6 patients with MTC and lenvatinib as second-line treatment in two patients with progressive disease under sorafenib treatment.

**Results:**

During the follow-up of treatment (mean 13.7 ± 7 months, median 12 months, range 6-32), 5/29 (17.2%) patients presented rare adverse events. These rare adverse effects were: heart failure, thrombocytopenia, and squamous cell carcinoma during sorafenib therapy and squamous cell carcinoma and oophoritis with intestinal perforation during vandetanib treatment.

**Conclusions:**

About 3 to 5 years after the approval of MKI therapy, we learned that MKIs usually lead to adverse effects in the majority of patients. Although most of them are manageable, we still need to be aware of potentially serious and rare or unreported adverse effects that can be life-threatening.

## INTRODUCTION

The advent of multikinase inhibitor (MKI) therapy has led to a radical change in the treatment of patients with advanced thyroid carcinoma. The use of these drugs should be considered in patients with radioiodine refractory differentiated thyroid cancer (DTC) or medullary thyroid cancer (MTC) with locally progressive or distant metastatic disease, and/or symptomatic disease that cannot be managed with surgery or local approaches (1).

The profile of adverse events of these drugs is usually well known. They were reported in up to 90% of patients in the ZETA, DECISION, and SELECT studies (2-4). The adverse events that occurred in less than 5% of patients in the clinical trials were considered rare. In the ZETA study (2), acute heart failure, aspiration pneumonia, respiratory arrest, respiratory failure, and staphylococcal sepsis were described in patients on the vandetanib arm with a frequency of 2.3% in each case. Squamous cell carcinomas (SCCs) occurred in 3.4% of patients in the sorafenib group in the DECISION trial (3). Acute myeloid leukemia and bladder cancer were described in 2.1% of cases (3). Only one patient on the sorafenib arm died due to myocardial infarction attributed to the study drug (3). Rare adverse events reported in patients who received lenvatinib were: acute renal failure (any grade, 4.2%; grade ≥ 3, 1.9%), hepatic failure (grade ≥ 3, 0.4%), gastrointestinal fistula (any grade, 1.5%; grade ≥ 3, 0.8%), and posterior reversible encephalopathy syndrome (any grade, 0.4%; grade ≥ 3, 0) (4).

The aim of this manuscript is to communicate rare adverse events that occurred in the clinical setting due to the use of these drugs.

## SUBJECTS AND METHODS

The authors performed a retrospective case-series study. Out of 760 patients with thyroid cancer, followed up in our Division of Endocrinology, 29 (3.8%) received treatment with MKIs. All of them had progressive disease at the time of the MKI initiation. Sorafenib was prescribed as first-line treatment to 23 patients with DTC and as second-line treatment in one patient with advanced MTC. Vandetanib was indicated as first-line treatment in 6 patients with MTC and lenvatinib (which is currently available only under the compassionate use program in our country) as second-line treatment in two patients with progressive disease under sorafenib treatment.

All patients undergoing MKI therapy followed an assessment protocol including 12-lead electrocardiogram parameters, vital signs, clinical chemistry, hematology, and urinalysis performed at 1, 2, 4, 8, and 12 weeks and every 3 months thereafter. Adverse events were assessed using the National Cancer Institute's Common Terminology Criteria for Adverse Events (CTCAE, v5.0).

## RESULTS

**Figure 1:**
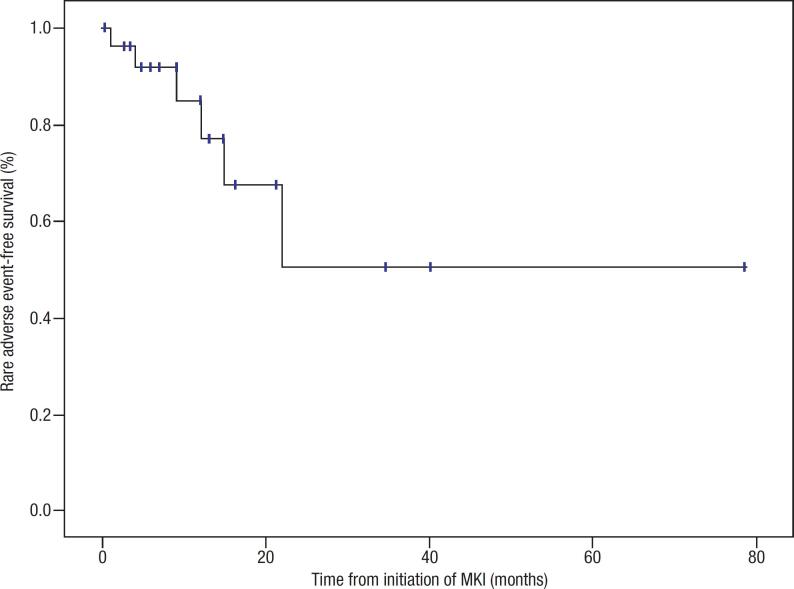
Kaplan-Meier curve showing the time between initiation of multikinase inhibitor (MKI) and development of rare adverse events.

The baseline characteristics of the 29 patients with thyroid cancer who received treatment with MKIs can be observed in Table 1. During the treatment follow-up (mean 13.7 ± 7 months, median 12 months, range 6-56), 5/29 (17.2%) patients presented rare adverse events. The characteristics and outcomes of these 5 patients are presented in Table 2. A Kaplan-Meier survival curve shows the time between initiation of MKIs and development of rare adverse events. The estimated median rare adverse event-free survival was not reached for our cohort of patients (Figure 1).

**Table 1:** Baseline characteristics of 29 patients with thyroid cancer who received treatment with multikinase inhibitors (MKIs)

Variable		(n = 29)
Gender		
	Male	7 (24.1%)
	Female	22 (75.9%)
Age at diagnosis of thyroid cancer (years)		
	Median (range)	53 (20-70)
Histology		
	Differentiated thyroid cancer	23 (79%)
	Medullary thyroid cancer	6 (21%)
First-line treatment		
	Sorafenib	23 (79%)
	Vandetanib	6 (21%)
Second-line treatment		
	Sorafenib	1 (3%)
	Vandetanib	0 (0%)
	Lenvatinib	2 (7%)
Duration of MKI treatment (months)		
	Median (range)	12 (6-56)

**Table 2:** Characteristics and outcomes of 6 patients with rare adverse events (AEs) occurring after multikinase inhibitor (MKI) treatment

	Age, gender	AE	Histology	MKI	Time after initiation before developing the AE (months)	Outcome	Mechanism that was probably involved
1	49, F	Cardiomyopathy (CHF)	Insular	Sorafenib	9	Improvement after suspension	Inhibition of VEGFR and PDGFR-β
2	67, F	Thrombocytopenia	Classic Papillary	Sorafenib	4	Recovery after withdrawal (800 mg/day) and reinitiation with 400 mg/day	Bone marrow affection secondary to RAI
3	64, M	SCC Diverticulitis and intestinal perforation	Medullary	Vandetanib Sorafenib	15 1.5	Surgery	Unknown Inhibition of VEGFR
4	70, M	4 SCC (4 surgical resections)	Classic Papillary	Sorafenib	22	Surgery	Activation of MAPK pathway
5	37, F	Oophoritis and intestinal perforation	Medullary	Vandetanib	12	Unilateral salpingooophorectomy	Inhibition of VEGFR

F: female; M: male; CHF: congestive heart failure; SCC: squamous cell carcinoma; VEGFR: vascular endothelial growth factor receptor; PDGFR-β: platelet-derived growth factor receptor β; MAPK: mitogen-activated protein kinase; RAI: radioiodine.

### Heart failure

A 49-year-old woman with a poorly differentiated insular thyroid cancer, metastatic to lungs and bones, was treated with sorafenib 800 mg/day. Nine months after sorafenib initiation, we observed a stable disease. She came to our hospital with severe heart failure. During this event, we excluded other common causes of heart failure. The electrocardiogram was also normal.

The initial ejection fraction (EF) before sorafenib prescription was 67%, which decreased to 25% when heart failure developed. One month after sorafenib withdrawal, the EF increased to 55%. The patient did not receive any other MKI treatment. However, she died 5 months later; it was a sudden death. This patient did not receive any other medication prior to the event that could have precipitated the heart failure or arrhythmia leading to sudden death.

### Thrombocytopenia

A 67-year-old woman developed dysphagia and dyspnea 63 months after initial treatment of papillary thyroid cancer. An 18-fluorodeoxyglucose (FGD) PET/computerized tomography showed progression of a local mass together with pulmonary metastases. Due to local unresectable disease, sorafenib 800 mg/day was then prescribed. Four months after sorafenib initiation, a grade 3 thrombocytopenia (25,000/mm^3^) developed and sorafenib was discontinued. The patient presented subconjunctival hemorrhage with spontaneous resolution. No other bleeding manifestations were detected. We performed a bone marrow study to exclude other causes of thrombocytopenia, but no other bone marrow cell linage reductions were observed.

Two weeks after sorafenib withdrawal, the platelet count was again in the normal range. Sorafenib was restarted at 400 mg/day, and no evidence of thrombocytopenia was observed after 30 months of treatment with a partial response to sorafenib treatment, according to RECIST 1.1 criteria.

### Squamous cell carcinoma (SCC)

Two patients developed SCC. The first patient was a 70-year-old man with a diagnosis of advanced and progressive papillary thyroid carcinoma with pulmonary metastases with partial response to sorafenib treatment. Twenty-two months after the MKI initiation, he developed SCC on his back, and 23 months later, three new lesions (two in the forearm and one in the ear) were also diagnosed as SCC. All of them were surgically removed. Currently, 56 months after sorafenib initiation, he continues the treatment at 400 mg/day dosage with partial response to treatment and with strict dermatologic control.

The second case is a 64-year-old patient with advanced MTC. He initially underwent cervical surgery three times (total thyroidectomy with central and left lateral lymphadenectomy, right lateral lymphadenectomy, and mediastinum lymphadenectomy). Three years after the initial treatment, cervical radiotherapy (4500 cGy) was indicated due to the presence of esophagus infiltration, but 5 years later, progression of mediastinum lymph nodes and hepatic and pulmonary metastases occurred. The largest pulmonary node was 2.2 cm in size and was located in the subpleural region of the right lower lobe. Thus, vandetanib 300 mg/day was prescribed. After 15 months of treatment, two skin tumors with rapid progression were detected, one over his chest and the other over his outer ear. The biopsy showed SCC. After 21 months of vandetanib treatment, he developed progressive disease and sorafenib 800 mg/day was prescribed as a second-line treatment. Forty days later, sorafenib was interrupted due to evidence of acute diverticulitis. The patient was then hospitalized due to a colonic perforation. Two months later, the patient died from generalized sepsis.

### Oophoritis and gastrointestinal perforation

A 37-year-old woman was diagnosed with MTC. Sixteen years after the initial diagnosis, she developed distant metastases in her liver and lungs associated with ectopic Cushing's syndrome. Therefore, vandetanib 300 mg/day was prescribed. After 1 month of treatment, she normalized the 24-hour urinary-free cortisol with improvement of signs and symptoms of Cushing's syndrome. She continued with the treatment without complications, but 1 year later, she presented at the emergency department with acute abdominal pain secondary to an ileal perforation. The pathological examination also described the presence of salpingo-oophoritis. Vandetanib was then stopped, and the Cushing's syndrome recurred immediately. She died 3 months after vandetanib withdrawal due to sepsis.

## DISCUSSION

A multidisciplinary team is always necessary for the prescription of MKIs in order to monitor and manage the adverse events that will inexorably occur in the majority of patients.

In the DECISION trial, the most commonly reported adverse effects in sorafenib treated patients were hand foot syndrome, diarrhea, alopecia, rash, weight loss, and hypertension (3). In the ZETA trial, diarrhea, rash, nausea, and hypertension occurred in more than 30% of patients receiving vandetanib (2).

Adverse effects occurring in less than 5% of patients in prospective trials are usually considered rare (2-4).

A serious and infrequent adverse event associated with MKI prescription is systolic and diastolic congestive heart failure (5). Patients may present with very dramatic symptoms of heart failure, as occurred with one of our patients (Case 1). This toxicity is not completely understood, but platelet-derived growth factor receptor-β (PDGFR-β) and vascular endothelial growth factor (VEGF) pathway inhibition (6) has been implicated as playing a role in the response to pressure overload-induced stress (7). The patient in Case 1 developed systolic congestive heart failure 7 months after the initiation of sorafenib therapy with improvement of the EF after sorafenib withdrawal.

The meta-analysis of Qi and cols. (8) including 10,553 patients with cancer treated with VEGFRMKIs showed an incidence of all-grade and high-grade congestive heart failure of 3.2% and 1.4%, respectively. Our patient eventually presented a sudden death after sorafenib withdrawal, which was probably not related to the MKI treatment. Sudden death was reported in only one case in the meta-analysis performed by Schutz and cols. (9).

Interestingly, a fatal case of cardiac failure after 14 months of vandetanib treatment was reported by Scheffel and cols. (10). In this report, myocardial infarction, coronary artery disease, Chagas's disease, and myocarditis was ruled out, and the postmortem histopathologic findings showed signs suggestive of chronic cardiotoxicity associated with VEGFR inhibitors (10).

Thrombocytopenia is another rare adverse effect presented in patients being treated with sorafenib. In hepatocellular carcinoma patients (SHARP study), the frequency of grade 3-4 thrombocytopenia was only 4% for those treated with sorafenib versus 1% for the placebo group (11), and in renal cell carcinoma patients (TARGET Study), this adverse effect occurred in only 1% of those treated with sorafenib versus 0% for the placebo group (12). In the DECISION trial, the frequency of thrombocytopenia was 18.4% for the sorafenib arm versus 9.6% in the placebo group. No grade 3 or 4 events were reported (3). The patient in Case 2 presented with grade 3 thrombocytopenia, which was solved with a dose reduction of the drug.

On the other side, secondary malignancies were reported in 4.3% of patients treated with sorafenib in the DECISION study, including 7 patients with skin SCC (3). We reported a patient who developed skin SCC during sorafenib treatment. Sorafenib seems to promote keratinocyte alteration and proliferation through activation of the mitogen-activated protein kinase path way and BRAF/CRAF heterodimerization, with subsequent activation of CRAF (13). Contrary to sorafenib, vandetanib was not associated with skin SCC. Vandetanib works by blocking RET, VEGFR-2, VEGFR-3, and EGFR (14). Indeed, the inhibition of EGFR and VEGFR has been implicated as a therapeutic target for head and neck SCC (15). However, although we do not believe this is the case, the possibility that the skin SCC in the patient from Case 5 was related to the vandetanib treatment cannot be completely excluded.

Finally, we reported the case of a patient who presented an intestinal perforation and salpingo-oophoritis during vandetanib treatment. The risk of gastrointestinal perforation has been associated with VEGFR inhibition, leading to vasoconstriction and thrombosis (16). However, in a systematic review including 300 patients under vandetanib treatment, the use of VEGFR tyrosine kinase inhibitors did not significantly increase the risk of gastrointestinal perforation in comparison with controls (OR 2.99, 95% CI 0.85-10.58, p = 0.089) (16).

In conclusion, we present 5 case reports of patients who developed rare (less than 5% in terms of frequency in clinical trials) adverse effects under sorafenib (n = 3) and vandetanib (n = 2) treatment.

About 3 to 5 years after their approval, we have learned that these drugs usually lead to adverse events in the majority of patients. Although most of them are manageable, we still need to be aware of potentially serious and rare or unreported adverse effects that can also be life-threatening. The rapid contact with the treating medical team is of vital relevance to prevent worse outcomes.
